# Neurogenic mechanisms for locomotor-respiratory coordination in mammals

**DOI:** 10.3389/fnana.2022.953746

**Published:** 2022-07-28

**Authors:** Laurent Juvin, Eloïse Colnot, Grégory Barrière, Muriel Thoby-Brisson, Didier Morin

**Affiliations:** ^1^University of Bordeaux, Centre National de la Recherche Scientifique, Institut de Neurosciences Cognitives et Intégratives d'Aquitaine, Unité Mixte de Recherche 5287, Bordeaux, France; ^2^Department of Health, Safety & Environment, Bordeaux Institute of Technology, Bordeaux, France

**Keywords:** breathing rate modulation, locomotor-respiratory coupling, neural network interactions, lumbar glutamatergic neurons, limb proprioceptive inputs

## Abstract

Central motor rhythm-generating networks controlling different functions are generally considered to operate mostly independently from one another, each controlling the specific behavioral task to which it is assigned. However, under certain physiological circumstances, central pattern generators (CPGs) can exhibit strong uni- or bidirectional interactions that render them closely inter-dependent. One of the best illustrations of such an inter-CPG interaction is the functional relationship that may occur between rhythmic locomotor and respiratory functions. It is well known that in vertebrates, lung ventilatory rates accelerate at the onset of physical exercise in order to satisfy the accompanying rapid increase in metabolism. Part of this acceleration is sustained by a coupling between locomotion and ventilation, which most often results in a periodic drive of the respiratory cycle by the locomotor rhythm. In terrestrial vertebrates, the likely physiological significance of this coordination is that it serves to reduce the mechanical interference between the two motor systems, thereby producing an energetic benefit and ultimately, enabling sustained aerobic activity. Several decades of studies have shown that locomotor-respiratory coupling is present in most species, independent of the mode of locomotion employed. The present article aims to review and discuss mechanisms engaged in shaping locomotor-respiratory coupling (LRC), with an emphasis on the role of sensory feedback inputs, the direct influences between CPG networks themselves, and finally on spinal cellular candidates that are potentially involved in the coupling of these two vital motor functions.

## Coordination of locomotor and respiratory rhythms

**Figure 1:**
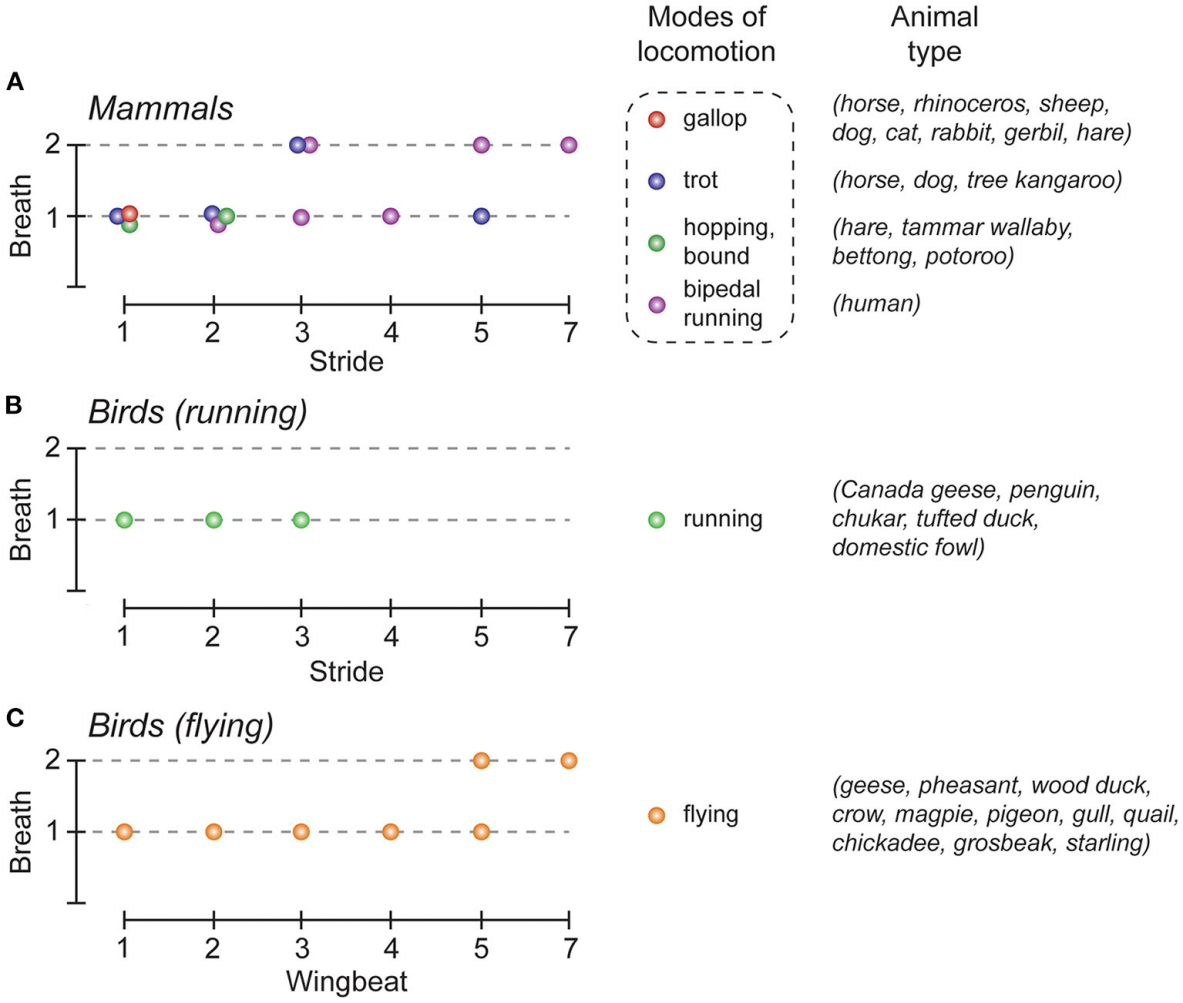
Locomotor-respiratory coupling (LRC) ratios in the animal kingdom. **(A–C)**, Graphical representation of the most common LRC ratios observed in mammals **(A)**, and birds **(B,C)**. The colored dots indicate the LRC ratios commonly observed, depending on the mode of locomotion used, in mammals (red, gallop; blue, trot; green, hopping/bond; purple, bipedal running), and birds (green, running; orange, flying). Note that for the same gait either one (e.g. gallop) or several (e.g., trot) LRC can be observed. Adapted from Boggs (2002); Stickford and Stickford (2014).

Breathing is a continuous rhythmic motor activity that maintains the homeostasis of blood gas tension. Its level of activity is thus finely tuned when body homeostasis is challenged, such as during physical effort. First, at the onset of exercise, breathing frequency increases in order to match or to anticipate the subsequent increase in energy consumption (Krogh and Lindhard, 1913; Dejours, 1959). Secondly, and in the specific context of locomotor activity, walking or running at increasing speeds results in an increase in respiratory frequency rates. Although the breathing frequency can be progressively and continuously adjusted during locomotion through the combined activation of peripheral and central chemoreceptors and mechanoreceptors located in joints and muscles, data collected across the vertebrate phylum have shown that specific coordination patterns between locomotor and respiratory activities can also occur in several species (Stickford and Stickford, 2014). This phenomenon, reported for fish, birds and mammals (Bramble and Carrier, 1983) is referred to as locomotor-respiratory coupling (LRC). One typical example of LRC is the 1:1 coupling that preferentially emerges in most mammals, notably during gallop (Figure 1). In this case, each locomotor cycle is associated with a coordinated respiratory motor sequence. LRC can also be favored by the contribution of multiple factors including external stimuli such as auditory cues (Bernasconi and Kohl, 1993) and training (Bramble and Carrier, 1983). But in some species, the synchronization of breaths to strides can be either absent [for example in adult mice (Hérent et al., 2020)], or differently expressed, as is the case in running or flying birds. Therefore, many different harmonic couplings (2:1, 3:1, 4:1, 5:2, etc.) have been reported in birds during flight (Butler and Woakes, 1980; Funk et al., 1992b; Boggs et al., 1997), and also in running quadrupeds and humans (Bechbache and Duffin, 1977; Bramble and Carrier, 1983; Perségol et al., 1991; Banzett et al., 1992; Bernasconi and Kohl, 1993). Finally, and regardless of the different coupling patterns that may occur, LRC has been suggested to produce a number of important physiological benefits by reducing biomechanical interferences between the respiratory and locomotor effector systems in order to assist breathing from forces produced during locomotion (Bramble and Carrier, 1983; Brown, 1999) or by decreasing the energetic cost of lung ventilation (Funk et al., 1992a). It has also been proposed that in running humans, LRC may reduce work in breathing by minimizing fatigue in the respiratory muscles that are essential for endurance aerobic activity (Daley et al., 2013).

### Biomechanical mechanisms

LRC can be ascribed to several underlying, and possibly redundant, mechanisms, the respective contributions of which may vary between animal species and might operate in parallel. Among these mechanisms, the biomechanical characteristics of the individual can be involved. Firstly, the “visceral piston” theory has been proposed to explain the 1:1 LRC observed in galloping quadrupeds. This idea proposes that the mechanical forward/backward movement of the internal organs during galloping physically affects movement of the diaphragm and contributes to the emergence of a 1:1 coupling between the locomotor and respiratory cycles (Bramble and Carrier, 1983; Young et al., 1992; Bramble and Jenkins, 1993). A similar mechanism could also operate in hopping animals that also express such coupling. Secondly, 1:1 LRC could result from changes in volume and pressure within the rib cage that occur when ground contact is made in each cycle. In galloping mammals, for example, the impact of the forelimbs with the ground results in an external loading on the thorax that compresses the rib cage. The resultant decrease in thoracic volume and the accompanying increase in pulmonary pressure thereby facilitates exhalation (Bramble and Carrier, 1983; Boggs, 2002). Finally, it has been postulated that 1:1 coupling could also emerge from changes in volume and pressure within the thoracic cavity associated with dorsiflexion of the lumbo-pelvic region. Mainly observed in fast running mammals such as the cheetah, the alternating lumbosacral flexions/extensions contribute to the rhythmic displacement of the visceral mass, which in turn facilitates exhalation and inspiration, respectively (Bramble, 1989; Stickford and Stickford, 2014).

### Neurogenic mechanisms

**Figure 2:**
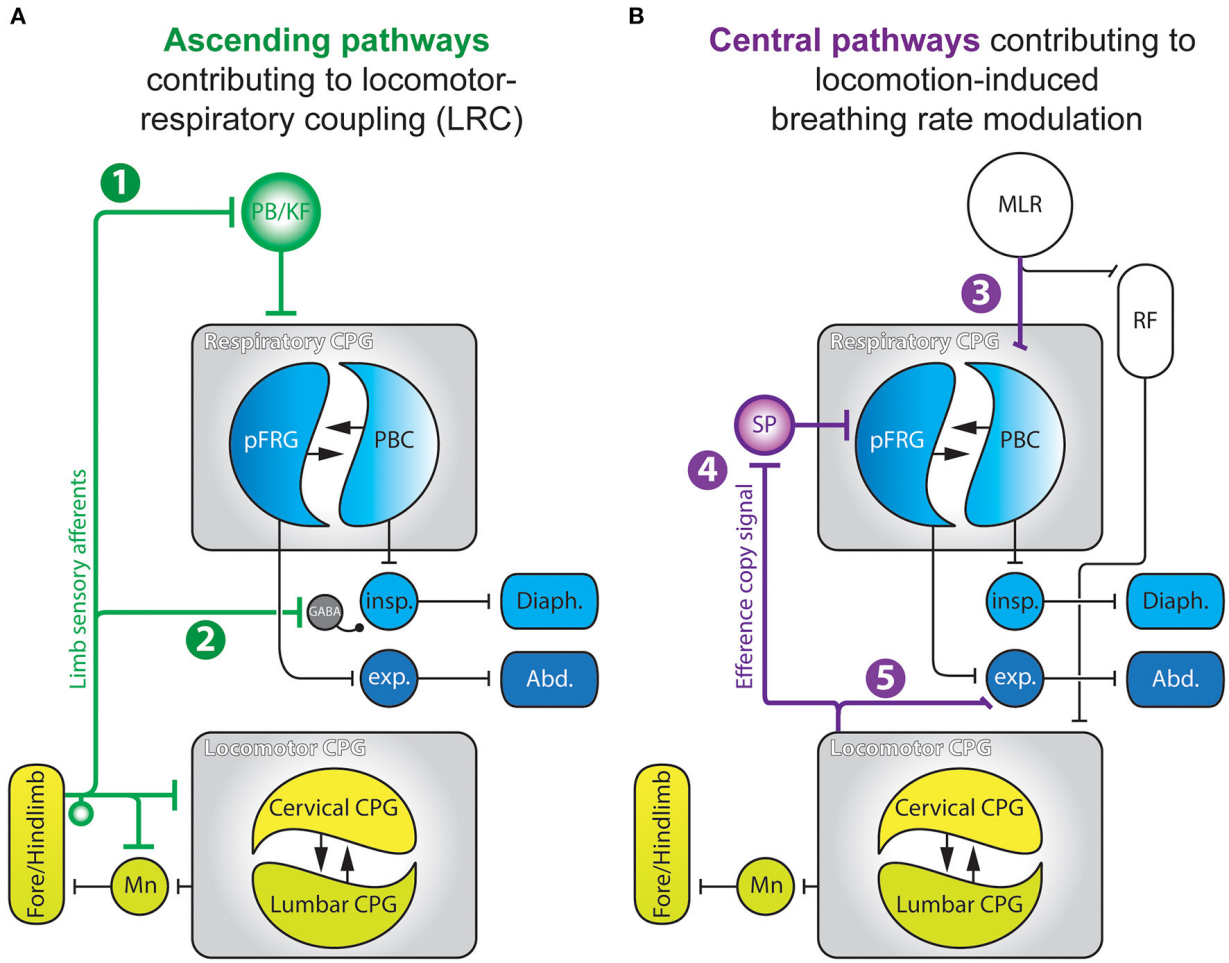
Summary schematics of the neurogenic pathways contributing to locomotor-respiratory coupling **(A)** and locomotion-derived breathing rate modulation **(B)**. 1, Ascending projections from limb sensory afferents to the PB/KF complex; 2, Pathway enabling spinal proprioceptive afferents to modulate the excitability of spinal inspiratory motoneurons *via* a GABA-releasing relay; 3, Parallel descending drives from the MLR to the spinal locomotor CPG and to the PBC; 4, Efference copy signals conveyed by ascending glutamatergic projections from the spinal locomotor CPG to respiratory CPG networks through an SP-releasing relay; 5, Propriospinal pathway enabling the locomotor CPG to modulate activity of spinal expiratory neurons. Abd., abdominal muscles; CPG, central pattern generator; Diaph., diaphragm; exp., expiratory neurons; insp., inspiratory neurons; MLR, mesencephalic locomotor region; Mn, motoneurons; PBC, preBötzinger Complex; PB/KF, parabrachial/Kölliker-Fuse nucleus; pFRG, parafacial respiratory group; RF, reticular formation; SP, substance P. Adapted from Morin and Viala (2002); Giraudin et al. (2008, 2012); Le Gal et al. (2016, 2020).

The above biomechanical mechanisms cannot explain either the emergence of harmonic LRC (e.g., 2:1, 3:2, 4:1, etc.) observed in birds and bipeds, or the progressive changes in the breathing rhythm that is observed during or even before the onset of locomotor episodes (Tobin et al., 1986; Gravel et al., 2007), implying that other mechanisms are also involved (Viala, 1997). On the one hand, neurogenic coupling processes, mainly involving feedforward mechanisms (Gariépy et al., 2010, 2012), have also been proposed to play a critical role in the regulation of breathing frequency during exercise. Indeed, feedforward pathways originating from the hypothalamus (Eldridge et al., 1981; Horn and Waldrop, 1998), the mesencephalic locomotor region (Horn and Waldrop, 1998; Ryczko and Dubuc, 2013; Opris et al., 2019), or medullary structures (Romaniuk et al., 1994), are likely to simultaneously influence the level of excitability of both the respiratory and locomotor rhythm generating networks. On the other hand, direct interactions between neuronal groups controlling locomotor and respiratory activities can also account for LRC. For instance, generation of the rhythmic locomotor and respiratory motor patterns both rely on so-called central pattern generator networks (CPG) located in the spinal cord (Viala and Vidal, 1978; Yamaguchi, 1992; Cazalets et al., 1995; Kiehn and Kjaerulff, 1996; Ballion et al., 2001; Kiehn, 2016) and the brainstem (Smith et al., 1991; Gray et al., 2001; Del Negro et al., 2018), respectively. Central pathway-mediated interactions between the locomotor and respiratory CPGs have been described in different animal models ranging from decerebrated paralyzed rabbits (Viala et al., 1987; Perségol et al., 1988; Corio et al., 1993), cats (Kawahara et al., 1989a,b), birds (Funk et al., 1992a,b), and *ex vivo* preparations of rodent central nervous systems (Morin and Viala, 2002; Le Gal et al., 2020). In all cases, experimental data have clearly indicated that the spinal locomotor CPGs can adjust the level of activity of the respiratory network through purely central mechanisms. In the following sections of this review, we will focus on the neurogenic mechanisms that subserve LRC (Figure 2A), or in a different manner, the ability of the locomotor neural system to regulate breathing frequency (Figure 2B).

## Peripheral feedback signals for locomotor-respiratory coupling

In different species, passive mobilization of the limbs modulates breathing activity and, in some cases, is able to entrain the breathing rhythm in a 1:1 manner (Bramble and Carrier, 1983; Viala, 1997; Boggs, 2002). During locomotion, limb sensory receptors are rhythmically activated (Prochazka and Gorassini, 1998a,b) and play an important role in the transition between the swing and stance phases of each movement cycle (Orlovsky et al., 1999; Duysens et al., 2000). This afferent control of the locomotor pattern timing is mediated through a direct feedback action on the spinal locomotor CPGs themselves (Conway et al., 1987; Kiehn et al., 1992; Sqalli-Houssaini et al., 1993; Perreault et al., 1995; Iizuka et al., 1997; Pearson et al., 1998; Schomburg et al., 1998; Juvin et al., 2012). Interestingly, fore and hindlimb sensory afferents also have access to the respiratory CPG where they are also likely to influence the latter's operation (Figure 2A) (Iscoe and Polosa, 1976; Bramble and Carrier, 1983; Palisses et al., 1988; Funk et al., 1992a). Indeed, passively-activated or electrically-stimulated limb sensory pathways are able to reset and entrain respiratory activity in a 1:1 manner in a large array of species and experimental preparations (Iscoe and Polosa, 1976; Bramble and Carrier, 1983; Funk et al., 1992a; Morin and Viala, 2002; Potts et al., 2005; Giraudin et al., 2008, 2012). For instance, in an *in situ* preparation of young rats (Potts et al., 2005), electrical stimulation of somatic afferents can reset the respiratory pattern cycle through a mechanism that does not rely on an intercalated intraspinal reflex-like mechanism, but rather *via* a direct action on the respiratory CPG itself. This was indicated by the finding that such resetting induced by somatic afferents involves the generation of a complete respiratory-like pattern, comprising the pre-inspiratory, inspiratory and expiratory phases of each cycle (Giraudin et al., 2008). In addition, repeated sensory stimulations are able to entrain the respiratory rhythm in a 1:1 manner, albeit within a specific range of stimulation frequencies (from 0.2 up to 0.4 Hz), which would not be expected from the involvement of a reflex-like mechanism (Potts et al., 2005). Similar results were reported from an *ex vivo* brainstem/spinal cord preparation of the newborn rat, where rhythmical electrical stimulation of spinal dorsal roots resets and entrains the ongoing respiratory rhythm over a limited range of stimulus frequencies [0.125 up to 0.25 Hz, (Morin and Viala, 2002)]. Moreover, this entrainment seems to be phase-dependent, since respiratory rhythm resetting appears to be facilitated when activation of limb somatic afferents are applied during expiration (Morin and Viala, 2002; Potts et al., 2005), producing a significant increase in the firing rate of medullary expiratory neurons (Potts et al., 2005). When stimulated, therefore, the somatic afferents cause initiation of inspiration *via* the initial activation of expiratory neurons. This respiratory phase transition, termed an “inspiratory on-switching” mechanism operating in the ponto-medullary region, relies strongly on the involvement of the parabrachial/Kölliker-Fuse (PB/KF) nucleus (see Figure 2A, pathway 1) (Cohen, 1971; Miura and Takayama, 1991; Chamberlin and Saper, 1994; Dutschmann and Herbert, 2006; Mörschel and Dutschmann, 2009). Indeed, several findings have indicated that the integrity of the PB/KF nucleus is necessary for respiratory entrainment during LRC. Firstly, calcium-imaging revealed that a cell population located within the PB/KF elicits calcium transients in response to individual spinal dorsal root stimulations (Giraudin et al., 2012). However, a reversible pharmacological inhibition of the PB/KF complex (Potts et al., 2005) or its bilateral lesion (Giraudin et al., 2012), prevent both the resetting and entrainment of the respiratory activity induced by limb sensory pathway stimulation. Although the exact role of the PB/KF complex in LRC remains open to debate, this pontine relay could be of primary importance during locomotion in gating out inappropriately timed sensory information resulting from antagonistic (flexor/extensor) movements of each of the four limbs. Such filtering would therefore ensure that the remaining ascending signals provide effective descending commands sent to medullary interneurons engaged in the cycle-by-cycle regulation of respiratory rhythmogenesis. The excitability of medullary respiratory neurons (Potts et al., 2005) and, ultimately, phrenic cervical motoneurons (Figure 2A, pathway 2) (Morin and Viala, 2002) would be modulated in such a way as to “prepare” these cell ensembles for responding preferentially to the premature respiratory command triggered by the spinal sensory afferent activation.

Finally, the exact nature and relative contributions of the different sensory afferent populations to the operation of the respiratory CPGs still remain uncertain, but virtually all types of fibers could be involved (Bruce et al., 2019). So far, small myelinated and non-myelinated fibers from groups III and IV muscle afferents have been found to contribute to the modulation of respiratory activity (Senapati, 1966; Decherchi et al., 2007; Amann et al., 2010). In contrast, the contribution of Ia and Ib primary afferents still remains a matter of debate in light of contradictory observations reported in the literature, with studies supporting (Bessou et al., 1959; Koizumi et al., 1961; Senapati, 1966; Carcassi et al., 1983; Morin and Viala, 2002; Potts et al., 2005; Giraudin et al., 2008, 2012) and others excluding their contribution (Waldrop et al., 1984). Further work is therefore needed to unravel whether these discrepancies arise from significant differences between experimental protocols or to species differences in the relative contributions of the different afferent populations.

## Central feedforward influences for breathing rate modulation

Moving at low or moderate pace triggers an elevation of the breathing frequency which is not directly coupled to the locomotor cycle frequency (Kawahara et al., 1989a; Hérent et al., 2020), especially in humans where LRC is observed during running but only sporadically during walking (Hill et al., 1988). This is also the case in the decerebrated cat, for instance, during locomotor episodes induced by stimulation of the mesencephalic locomotor region (MLR). At moderate locomotor pace, such as during trotting, the frequency of breathing activity increases, but LRC emerges only at the most rapid locomotor pace, especially during galloping (Kawahara et al., 1989a). The elevation of the ventilatory rate observed at the initiation of locomotion, or even before the onset of the physical effort (Tobin et al., 1986; Gravel et al., 2007) cannot only rely on sensory feedback mechanisms, including peripheral chemoreception, since blood gas homeostasis is maintained at the onset of exercise (Mateika and Duffin, 1995; Haouzi et al., 1997). On this basis, therefore, the anticipatory respiratory response must rely on central feedforward mechanisms that are different from those involved in direct locomotor–respiratory coupling (Figure 2B) (Tobin et al., 1986; Bell, 2006; Gravel et al., 2007; Gariépy et al., 2010). Several brain regions involved in the initiation of locomotor episodes, including the hypothalamus (Eldridge et al., 1981; DiMarco et al., 1983; Waldrop and Iwamoto, 2006), the MLR (Gariépy et al., 2012), and the ponto-medullary reticular formation have been shown to produce modulation of the breathing frequency. In the unanesthetized decorticated cat, electrical stimulation of the sub-thalamic locomotor region (SLR) triggers episodes of locomotion. Significantly, the initiation of locomotor movement is preceded by a rapid modulation of the breathing frequency, with breathing amplitude correlated to the intensity of the locomotor activity (Eldridge et al., 1981). These anticipatory responses were also observed in the paralyzed cat where phasic sensory feedback from limb afferents was lacking due to the absence of muscle contractions (Eldridge et al., 1981). In the cat and the lamprey, stimulation of the MLR also triggers a rapid elevation of respiratory rate concomitant with the initiation of locomotor activity (Figure 2B, pathway 3) (Kawahara et al., 1989b; Gariépy et al., 2012). In the lamprey model furthermore, these authors reported that the MLR projects directly to the respiratory CPG network, and that stimulations of the MLR that fail to trigger locomotion can nonetheless increase the respiratory frequency. Thus, an activation of supra-spinal locomotor structures is capable of eliciting an integrated response in both the respiratory and locomotor systems. Such a feedforward mechanism would serve to adapt ongoing respiratory activity in order to anticipate and minimize the metabolic changes induced by locomotion. However, the nature and relative contribution of the different locomotor regions in the feedforward regulation of the respiratory network remains to be fully described.

Other CNS regions are also involved in the modulation of the respiratory CPG network, especially the spinal cord where the locomotor CPGs controlling the limbs are located. In the *ex vivo* neonatal rat brainstem-spinal cord preparation, both locomotor and respiratory motor outputs can be recorded from specific spinal motor roots (Smith and Feldman, 1987). In this isolated preparation, the motor commands normally sent to the limb muscle effectors were monitored by electroneurograms, and the recorded activities referred to as “fictive” respiration and locomotion due to the inevitable absence of effective movement. In this preparation, we have shown that the specific activation of the spinal locomotor CPGs induces a rapid increase in the frequency of spontaneous fictive respiration (Morin and Viala, 2002; Le Gal et al., 2014). Similar to the activation of the supra-spinal locomotor regions, a slowly increasing pharmacological stimulation of the spinal locomotor CPGs evokes an increase in fictive respiration frequency that precedes the onset of fictive locomotion (Le Gal et al., 2020). Moreover, both the lumbar and cervical locomotor CPGs controlling the hindlimbs and forelimbs, respectively (Cazalets et al., 1995; Ballion et al., 2001) can modulate respiratory activity (Le Gal et al., 2020), with this ascending central influence being integrated at the level of the parafacial respiratory group (pFRG) via an intercalated substance P-releasing relay (Figure 2B, pathway 4). Both the bilateral removal of the pFRG or the pharmacological blockade of brainstem substance P receptors abolishes the ascending modulation of the respiratory network by the spinal locomotor CPGs (Le Gal et al., 2014). However, blocking spinal thoracic circuitry interposed between the brainstem respiratory network and the lumbar locomotor CPG does not affect the ascending modulatory action, indicating the involvement of long ascending projection pathways originating in the lumbar spinal area (Le Gal et al., 2020). In order to identify the nature of the neurons involved in this modulation, we performed optogenetic stimulation of glutamatergic Vglut2-positive neurons previously shown to participate in the generation of fictive locomotion (Hägglund et al., 2010). Using this stimulus method, we found a concomitant increase in the rate of fictive respiration (Le Gal et al., 2020). Moreover, spinal V2 interneurons themselves are likely to be a source of the long ascending projections to the respiratory CPG, since firstly, they are known to interconnect the lumbar and cervical locomotor networks to strengthen interlimb coordination (Ruder et al., 2016), and second, they are rhythmically active during fictive locomotion (Dougherty and Kiehn, 2010; Zhong et al., 2010; Dougherty et al., 2013). Additionally, a subset of glutamatergic V2 neurons (V2a) have been found to project from the spinal cord to the brainstem where they convey an internal efference copy of the ongoing motor command sent to the forelimb muscles during skilled reaching (Brockett et al., 2013; Azim et al., 2014).

Finally, the spinal locomotor CPGs also influence respiratory motor output at the level of the spinal cord itself. In the isolated brainstem–spinal cord preparation from neonatal rat, the activity of expiratory, but not inspiratory, motoneurons and interneurons is rhythmically modulated during fictive locomotion (Figure 2B, pathway 5) (Le Gal et al., 2016). Similar results were obtained when recording simultaneously from the spinal ventral roots, motor nerves and limb and trunk muscles in semi-intact preparation of newborn rat (Iizuka et al., 2022). From a functional perspective, this latter observation is relevant to the existence of bi-functional muscles, as is the case for the abdominal muscles, which participate in both respiration and locomotion (Saunders et al., 2004; Iizuka et al., 2022). Indeed, axial musculature, which is involved in expiration in mammals (Koterba et al., 1988; De Troyer et al., 1990; Deban and Carrier, 2002), is also engaged in locomotion (Grillner et al., 1978; Puckree et al., 1998; Deban and Carrier, 2002; Reilly et al., 2009). It has been proposed that this influence of the lumbar locomotor generator on expiratory neurons that control trunk movements could induce bending of the spine, thereby generating a forward displacement of the pelvis, which is known to facilitate exhalation.

## Concluding remarks

The spinal and supraspinal circuits involved in the interactions between locomotor and respiratory functions are distributed over several CNS regions, making their overall understanding a challenging task. Clearly, the neural networks and pathways described here represent only a portion of the circuitry involved and, besides mechanical constraints and neurogenic control processes, metabolic conditions also influence locomotor-respiratory coordination. Although it is very likely that the experimental observations thus far obtained using reduced CNS preparations have only partially revealed the mechanisms engaged in the coordination between the respiratory and locomotor networks, these data have nonetheless provided important insights into the functional relationships between these two mammalian motor functions. Already initiated in the newborn rodent (Le Gal et al., 2020), efforts to identify the cell types involved in this coupling should now be further pursued, both by using *ex vivo* preparations but also in the behaving animal, in particular by taking advantage of the development of transgenic models and advanced experimental tools to monitor and analyze motor activities. Ultimately, a precise knowledge of the neural mechanisms and cellular pathways involved in the coordination between these two vital behaviors should have important implications for developing rehabilitation procedures after an incomplete spinal cord injury (Sutor et al., 2022).

## Author contributions

LJ, EC, GB, MT-B, and DM wrote and edited the manuscript text. All authors contributed to the article and approved the submitted version.

## Funding

This work was supported by an Equipe FRM (Fondation pour la Recherche Médicale) funding (DEQ20170336764) to MT-B.

## Conflict of interest

The authors declare that the research was conducted in the absence of any commercial or financial relationships that could be construed as a potential conflict of interest.

## Publisher's note

All claims expressed in this article are solely those of the authors and do not necessarily represent those of their affiliated organizations, or those of the publisher, the editors and the reviewers. Any product that may be evaluated in this article, or claim that may be made by its manufacturer, is not guaranteed or endorsed by the publisher.
